# Climate Change and Hydropower Impacts on Habitat Suitability of Endangered Schizothoracinae Fishes in the Qinghai‐Xizang Plateau

**DOI:** 10.1002/ece3.73786

**Published:** 2026-06-18

**Authors:** Yan Zhou, Qize Zheng, Fei Liu, Zhaofang Han, Hongxin Liu, Xue Wang, He Gao, Hongbo Pan, Yangyang Li, Jishun Ma, Chaowei Zhou, Yao Li, Haiping Liu

**Affiliations:** ^1^ School of Ecology and Environment, Xizang University Lhasa China; ^2^ School of Life Sciences, Southwest University Chongqing China; ^3^ College of Fisheries, Southwest University Chongqing China; ^4^ School of Geographical Sciences, Southwest University Chongqing China

**Keywords:** climate change, habitat suitability, hydropower development, maximum entropy model, Schizothoracinae fish, the Yarlung Zangbo River

## Abstract

Climate change and human disturbance threaten freshwater biodiversity, particularly endangered species on the Qinghai‐Xizang Plateau. *Oxygymnocypris stewartii* , *Schizothorax macropogon* , and *Schizothorax waltoni* (*Schizothorax* spp. hereafter) are Class II Protected Species in China, indigenous to the Qinghai‐Xizang Plateau, and primarily distributed in the middle and upper Yarlung Zangbo River (MUR). Based on species distribution points, the Maximum Entropy (MaxEnt) model was used to simulate potentially suitable habitats and identify key environmental factors for *Schizothorax* spp. Ecologically suitable areas were reconstructed using a kernel density estimation (KDE) algorithm that incorporates hydropower plants, and future distribution dynamics were predicted under various climate scenarios. Results indicated that the flow accumulation and the mean temperature of the coldest quarter were the primary environmental factors affecting *Schizothorax* spp. For *Schizothorax* spp., the suitable habitat exhibited a non‐monotonic trend: it increased before the 2050s (up to 108.28% at 2050s‐SSP7.0), then turned downward by the 2070s (18.1% at 2070s‐SSP2.6). Moreover, habitat suitability sustained expansion at the upper reaches, with the rate of expansion increasing with the intensification of radiative forcing, and projected an increase in elevation at SSP7.0 and SSP8.5. At the local scale, however, hydropower plants pose a deterministic threat, causing permanent habitat loss of *Schizothorax* spp. in the middle and lower reaches. Overall, the model predicts that future climate‐driven events may lead to a spatial expansion of potentially suitable habitats for *Schizothorax* spp. However, due to limitations in hydrological connectivity and the unique characteristics of fish, these high‐altitude habitats face significant constraints. Enhanced conservation efforts in the Saga, Angren, and Miling basins of the MUR are recommended, along with continuous monitoring and early warning of changes in habitat suitability to protect the Schizothoracinae conservation areas.

## Graphical abstract

FA and Bio11 are key factors impacting Schizothorax species’ habitats. The Saga, Angren, and Miling counties were suitable areas considering hydropower. Schizothorax species will increase upriver and lose lower habitats in the future. Schizothorax species were exhibited an upstream movement trend in the future.

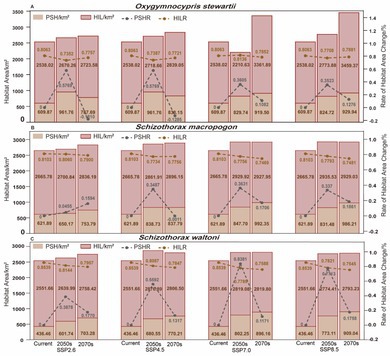

## 1 Introduction

Accumulating evidence indicates that climate change and human activities are key drivers of global biodiversity dynamics, altering natural habitats and significantly reshaping species' survival strategies and geographic distributions (Román‐Palacios and Wiens 2020; Barbarossa et al. 2021; Ghosh et al. 2024; Brodie et al. 2025). However, not all organisms, especially freshwater species, can quickly adapt to these habitat changes (Wiens 2016). Human activities frequently impact freshwater ecosystems, resulting in habitat fragmentation (e.g., construction of hydroelectric power stations) or habitat loss (e.g., agricultural development or urbanization), in turn reducing fish stocks, resources, and may even drive species extinction (Agostinho et al. 2008; Turgeon et al. 2019; Wu et al. 2019; Sor et al. 2023; Wang et al. 2023). Additionally, climate change is altering fish distribution patterns, with freshwater species migrating to higher‐elevation rivers or latitudes (Heino et al. 2009). For example, research tracking the geographic ranges of 15 stream fish species over 25 years revealed an average northward shift of 51 km and an upward elevation shift of 32.7 m (Hickling et al. 2006). Furthermore, climate change can facilitate the spread of invasive fish species by altering environmental conditions, expanding suitable habitats, and diminishing the competitive advantage of native species, ultimately promoting the colonization and dominance of exotics (Su et al. 2023; Di Febbraro et al. 2023; Ji et al. 2025). Therefore, developing effective conservation measures for freshwater fish, especially for rare and endangered native species, remains a critical challenge for ecologists.

Schizothoracinae fishes are the only natural group within the Cyprinidae family that have evolved to survive in the extreme environment of the Qinghai‐Xizang Plateau, and as key representatives of its biodiversity, they play a crucial role in maintaining ecosystem function and have attracted significant attention (He and Chen 2006; Deng et al. 2020; Du et al. 2024). Schizothoracinae fishes are among China's most threatened freshwater fish communities, with approximately 55% of the species facing severe survival crises due to environmental stressors in their habitats (Cao et al. 2024). Among them, *Schizothorax* spp. are all classified as Grade 2 nationally protected species in China. *O. stewartii* (Ng 2010a) and *S. macropogon* (Ng 2010b) are also ‘Near Threatened’ fish species on the IUCN checklist, while *O. stewartii* (Endangered) and *S. waltoni* (Near Threatened) are involved in *China's Red List of Biodiversity*‐Vertebrate Volume (2020) (Liu et al. 2025). Hence, identifying and planning suitable basins for the protected Schizothoracinae fish ecologically is an extremely urgent conservation priority.

The MUR, which is the headwater of the Brahmaputra River, serves as the primary habitat basin for *Schizothorax* spp., while the *O. stewartii* is found exclusively in this basin (Yang et al. 2010; Yang and Huang 2011; Liu, Ma, et al. 2020; Liu, Li, et al. 2021). Although this region contains plentiful fishery resources, the community composition remains simplistic, and the hydro‐ecosystem is notably vulnerable (Peng et al. 1995; Guo et al. 2018). Recently, the construction and operation of hydropower plants, leveraging the steep gradients in the watershed, have significantly promoted clean energy supplies, contributing markedly to achieving carbon neutrality. However, these changes in hydrological conditions have raised significant concerns about the sustainability of fish populations in this basin (Zhou et al. 2017; Li, Jin, et al. 2022; Li, Wang, et al. 2022; Liu et al. 2025). For instance, the research confirmed that habitat for the juvenile stages of the *Schizopygopsis younghusbandi* population was significantly decreased in both spring and winter (Luo et al. 2025). Furthermore, projections indicated that suitable habitats for *Glyptosternum maculatum* in the Yarlung Zangbo River basin could diminish by up to 20% under future warming conditions (Zheng et al. 2024). These findings emphasized the high sensitivity of the fish community in the alpine basins to environmental alterations and highlighted the variable nature of the ecosystem's health. Consequently, balancing the protection of fish community resources with sustainable energy development has emerged as a critical challenge in watershed health management.

The MaxEnt model is thought to be one of the most reliable models for predicting regional changes of species distribution, delivering authentic consequences with limited samples, and is extensively applied in the conservation ecology assessment of rare and threatened species (Phillips et al. 2006; Shao et al. 2023; Zheng et al. 2024; Bosso et al. 2025; Zhong et al. 2025). For freshwater ecosystems in South Korea, the habitat suitability of the exotic fish *Micropterus salmoides* under climate variability was successfully predicted, and influential environmental factors were assessed using the MaxEnt model (Mamun et al. 2018). Similarly, Shao et al. (2023) employed MaxEnt to predict optional habitat zones for Cyprinidae in the Pearl River basin and to simulate their hotspot distribution within the watershed. The distribution of fish in the Irrawaddy River was effectively evaluated by the MaxEnt model, providing a worthy strategy for watershed conservation (Li, Qin, et al. 2021). While research on the impacts of climate change and human activities on fish distribution has laid the groundwork, there remains a lack of fine‐scale studies necessary to formulate precise conservation measures for the MUR (Li et al. 2019; Liu et al. 2025). Therefore, this study used MaxEnt to identify potentially suitable habitats and then employed kernel density to quantify the spatial distribution pressure from hydropower stations. Through this two‐dimensional assessment framework, we conducted an in‐depth investigation into the effects of multiple stressors on habitat suitability for plateau‐endemic fish species in the future. We hypothesize that, under future climate scenarios, the area of suitable habitat for *Schizothorax* spp. may show an upward trend; however, due to habitat fragmentation and the species' unique growth characteristics, this may ultimately result in a decline in actual habitat suitability.

## 2 Materials and Methods

### 2.1 Study Area

**FIGURE 1:**
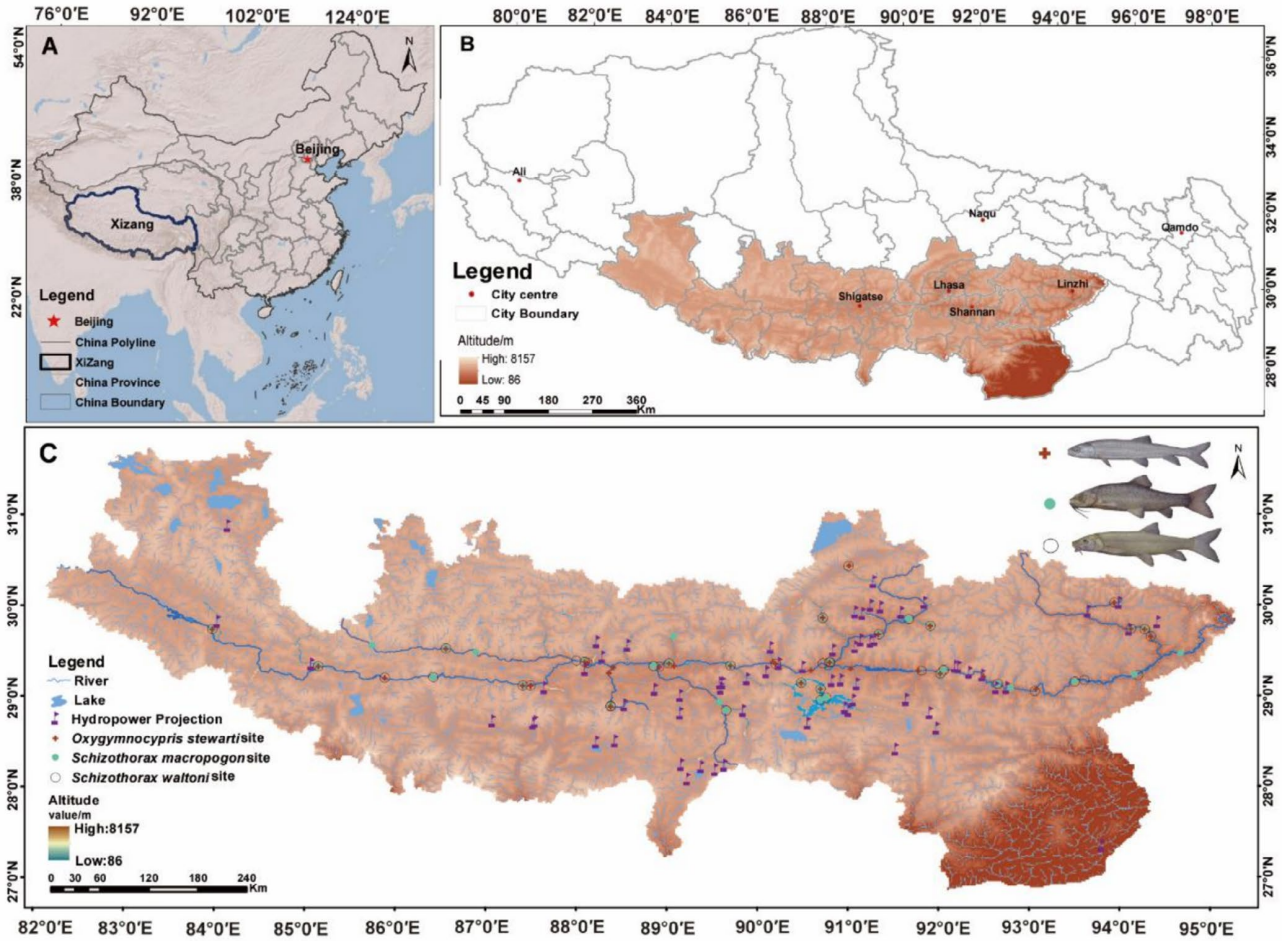
Location of the MUR and distribution of *Schizothorax* spp. in this study.

The Yarlung Zangbo River Basin is among the 36 global biodiversity hotspots (Figure 1A,B), celebrated for its remarkable ecological significance and high levels of faunal endemism (Myers et al. 2000). The basin supports 32 indigenous fish species, including 20 Schizothoracinae species (Liu et al. 2025). The Yarlung Zangbo River has a total drop of 5435 m, providing favorable conditions for hydropower development; at the same time, *Schizothorax* spp. are primarily located in the main and tributary sections of MUR (Figure 1C) (28°00′ N–31°16′ N, 82°00′ E–97°07′ E) (Peng et al. 1995; Liu et al. 2025).

### 2.2 Data Acquisition

#### 2.2.1 Fish Data Collection

The distribution coordinates of *Schizothorax* spp. (Figure 1C) were collected from field surveys (2019~2021) and publicly available sources, including China Knowledge (https://www.cnki.et), the Global Biodiversity Information Facility (https://www.gbif.org), and Fishbase (https://www.fishbase.se). Field surveys used gillnets and bottom traps. Three gillnet sizes were used: 1 cm mesh (30 × 0.8 m), 3 cm mesh (30 × 1.0 m), and 5 cm mesh (50 × 1.2 m); bottom traps had a mesh size of 0.2 cm (5 × 0.25 m × 0.25 m). All operations were conducted in accordance with regulations governing the management of laboratory animals and fisheries permits. To minimize injury and mortality among the fish, all captured individuals were immediately released back into the river section where they were caught after being recorded.

The total number of fish sites recorded was 45 for *Schizothorax* spp. (Table S1), with records from public sources for *O. stewartii* : 12, *S. macropogon* : 17, and *S. waltoni* : 14. These records represent the most comprehensive and verified collection for these rare alpine species in the MUR. Hence, despite potential sampling biases, all coordinate data were converted to a CSV format compatible with the MaxEnt model to support subsequent analysis.

#### 2.2.2 Hydropower Station Data

Hydropower plant locations were obtained from a geospatial database comprising about 100,000 reservoirs across China (https://doi.org/10.5281/zenodo.6984619), which provides detailed information on reservoirs, hydropower stations, and their geographic coordinates. In the study area, 64 hydropower projects were identified (Figure 1C).

#### 2.2.3 Environmental Data

1. Current environmental data were obtained. Based on previous literature on fish studies, four main categories of environmental elements were selected to explore the geographic distribution of *Schizothorax* spp. in the MUR (Frederico et al. 2014; Li, Song, et al. 2021; Zhang et al. 2021; Mu et al. 2022; Zheng et al. 2024). All selected factors were as follows: (1) Climate Data: 19 climate factors obtained from the WorldClim database web (https://www.worldclim.org/) with 1 km spatial resolution ratio; (2) Topographic Data: elevation and slope were gained from the Geospatial Data Cloud website (https://www.gscloud.cn/) with a 30 m spatial resolution ratio; (3) Riverscapes: Normalized Vegetation Index (NDVI) data were acquired with a 30 km spatial resolution ratio, reflecting vegetation cover status as a proxy for ecosystem productivity, and (4) Hydrological Data: flow accumulation data were got from the website of the EarthEnv (http://www.earthenv.org/).
2. Future environmental data were acquired. The four scenarios (SSP2.6, SSP4.5, SSP7.0, and SSP8.5 from the Beijing Climate Center Climate System Model 2 Medium Resolution (BCC‐CSM2‐MR)), based on the Sixth Coupled Model Comparison Program (CMIP6) in the WorldClim database, were used for the 2050s (2041–2060) and 2070s (2061–2080) periods, with a spatial resolution of 1 km. The four Shared Socioeconomic Pathways (SSPs) in the BCC‐CSM2‐MR model represent different climate scenarios by 2100, based on greenhouse gas (GHG) concentration levels and radiative forcing values. SSP2.6 means a low‐emission scenario in which the GHG concentration is significantly reduced, reaching net zero by 2050, leading to a 1.8°C increase by 2100. SSP4.5 represents a moderate‐emission scenario in which the emissions keep stable before gradually declining in the mid‐21st century but do not achieve zero, leading to a 2.7°C rise by 2100. SSP7.0 represents a medium‐to‐high‐emission scenario in which GHG emissions continue to rise, causing a 4.0°C increase by 2100. SSP8.5 represents a high‐emission scenario in which the emissions will roughly double by 2050, leading to a 4.4°C rise by 2100 (Jin et al. 2022). To maintain consistency across the timeline and ensure comparability of the models, future projections also incorporate unchanged datasets, including data on terrain and landform, grain resources, hydrology, and hydroelectric power plants. These scenarios have been verified to be well‐suited for simulating China's climate (Zheng et al. 2024; Wu et al. 2019).
3. Standardization and preprocessing. All environmental factors were standardized into a unified layer and resampled to a spatial resolution ratio of 1 km. A Pearson correlation analysis was used to mitigate multicollinearity among climatic variables (Figure S1) (Dormann et al. 2013). If the correlation coefficient is greater than 0.8 (|*r*| ≥ 0.8), retain the most relevant variables based on their percentage contribution to the initial model and exclude the others; sort the remaining variables by their percentage contribution and retain them. Then, the environmental variables were used in the final model. After correlation screening, a total of 4 key environmental variables were ultimately included in the MaxEnt model to predict *Schizothorax* spp.: 2 topographic factors (elevation, slope) and 2 hydrological and landscape factors (FA, NDVI). Climatic factors for *O. stewartii* prediction were 4 factors (Bio4, Bio5, Bio11, and Bio14), *S. macropogon* were 3 factors (Bio4, Bio11, and Bio14), and *S. waltoni* were 3 factors (Bio5, Bio11, and Bio14). The detailed statistical characteristics of these variables are presented in Table 1. Subsequently, all environmental raster data were converted to ASCII format for compatibility with MaxEnt modeling.

**TABLE 1:** Environmental variables final used to simulate the potential distribution of *Schizothorax* spp.

Variables	Fish species	Source of data	Resolution
Temperature Seasonality (Bio4)	*O. stewartii* , *S. macropogon*	http://www.worldclim.org	1 km
Max Temperature of Warmest Month (Bio5)	*O. stewartii* , *S. waltoni*	http://www.worldclim.org	1 km
Mean temperature of the coldest quarter (Bio11)	*O. stewartii* , *S. macropogon* , *S. waltoni*	http://www.worldclim.org	1 km
Precipitation of Driest Month (Bio14)	*O. stewartii* , *S. macropogon* , *S. waltoni*	http://www.worldclim.org	1 km
Flow accumulation (FA)	*O. stewartii* , *S. macropogon* , *S. waltoni*	http://www.earthenv.org	1 km
Altitude	*O. stewartii* , *S. macropogon* , *S. waltoni*	http://www.gscloud.cn	30 m
Slope	*O. stewartii* , *S. macropogon* , *S. waltoni*	http://www.gscloud.cn	30 m
NDVI	*O. stewartii* , *S. macropogon* , *S. waltoni*	http://www.gscloud.cn	30 m

### 2.3 MaxEnt Modeling Analysis

#### 2.3.1 Optimization of MaxEnt Model Parameters

The two most important parameters in the MaxEnt model are the repetition factor (RM) and feature class (FC); optimizing these parameters significantly improves model accuracy (Radosavljevic and Anderson 2014). The RM parameter is set within the range [0.1–4], with one RM value assigned for every 0.1 increment, resulting in a total of 40 RM values; FC has 5 options: Linear (L‐Linear features), Quadratic (Q‐Quadratic features), Product (P‐Product features), Hinge (H‐Hinge features), and Threshold (T‐Threshold features), resulting in 29 different combinations. Using the kuenm package in R (Cobos et al. 2019), prediction calculations were performed for 1160 different parameter models (40 RM values and 29 FC settings freely combined) using the MaxEnt model. R software selects the optimal results from all candidate models based on the following criteria: statistical significance and an omission rate below 5% (Cobos et al. 2019). It then uses the Akaike Information Criterion (AICc) to select the final recommended model(s) with a Delta AICc value less than 2. If more than one model is recommended, the model with the smallest Delta AICc value is selected as the final recommended model (R code in Supporting Information S2). The results showed that Model 1 had the smallest Delta AICc value (equal to 0), indicating that this model provided the best fit. The corresponding FC combination and RM were determined to be the optimal parameters, namely RM = 3 and FC combination H.

#### 2.3.2 Model Construction

The potential suitability zones for *Schizothorax* spp. were predicted using MaxEnt 3.4.4 software to model habitat suitability by integrating various environmental elements, such as climate, topography, and hydrology (Mu et al. 2022; Zheng et al. 2024). To ensure the scientific rigor of model development and to conduct rigorous independent validation, this study followed general guidelines for species distribution modeling by randomly dividing the distribution records for each species into a training set (75%) and a test set (25%). The training set was used to parameterize the model to capture the bioclimatic envelope characteristics of the species, while the test set served as an independent sample to evaluate the model's predictive performance in non‐training areas. To eliminate the bias caused by random data partitioning in a single run and enhance the model's robustness, the model was run 10 times using 10‐fold cross‐validation, and final result was calculated as the mean and standard deviation across all runs. The contribution of environmental factors was assessed using the Jackknife test, and the suitable ranges for key environmental factors were determined based on the species' response curves. The model outcome was produced in logistic format. For model accuracy evaluation, this study employed a combined assessment using the area under the receiver operating characteristic (ROC) curve (AUC) and the true skill score (TSS) (Lobo et al. 2008). The AUC ranges from 0 to 1; the closer the value is to 1, the better the model performs (Phillips et al. 2004). The TSS metric further quantifies the model's discriminatory power at a specific threshold, ensuring that the evaluation results are not influenced by species prevalence (Lobo et al. 2008).

The SDMtools tool was utilized to calculate distribution centroids for different scenarios and periods. By loading the SDMtoolbox toolkit into ArcGIS, the “Distribution Change Between Binary SDMs” toolkit was applied to count centroids under various scenarios. Additionally, the “Centroid Changes (Lines)” toolkit was used to assess shifts in the center of mass of Splittail's distribution, revealing overall trends in suitable habitat regions. Although calculating center‐of‐mass displacement using SDMtoolbox is a common method for assessing habitat shifts, it is important to recognize its limitations within the constraints of river networks (Altermatt 2013). Hence, the center‐of‐mass displacement reported in this study should be interpreted as the spatial evolution of species‐suitable habitat at the regional scale, rather than as specific dispersal pathways.

#### 2.3.3 Planning Ecologically Suitable Areas for *Schizothorax* spp.

Although MaxEnt performs well at predicting distributions, its application in freshwater systems has neglected river network connectivity (Domisch et al. 2015; Altermatt 2013). To address this limitation and scientifically assess hydropower impacts, this study developed an integrated framework that proceeds in defined steps. First, MaxEnt is used to delineate ecologically suitable habitats. Next, it is coupled with the estimated spatial distribution of hydropower plants by KDE (Bosso et al. 2025; Zheng et al. 2024). Using spatial overlay analysis, we reconstruct planning ecologically suitable areas. Using this as a baseline, the dynamic changes in suitable habitats for this species under future climate scenarios were predicted. Although KDE is often used to measure the spatial intensity of anthropogenic disturbance, its isotropy assumption does not hold in river systems with strong longitudinal constraints (Cote et al. 2009). In this study, KDE serves as a macro‐scale indicator of hydropower development pressure, rather than a substitute for hydrological connectivity models. Finally, a response matrix is constructed by combining habitat suitability and development pressure via superposition analysis to reflect complex cumulative impacts.

KDE is a nonparametric spatial prediction method for calculating the density of spatial elements within their periphery neighborhood. For a given point in space, the distribution of its attributes is defined within a circle of radius ℎ (threshold). To objectively reflect the spatial clustering characteristics of hydropower stations, this study employed Silverman's Rule of Thumb to automatically optimize the bandwidth (Silverman 2018). Based on algorithmic calculations, the optimal bandwidth h for the MUR was determined to be 0.27° (approximately 30 km) (Zheng et al. 2024). The distribution pattern decreases with distance, with the highest density at the center, and the density approaches zero at the limiting distance. The sum of the integrals of densities in the threshold scope represents the property value of the core point. In this study, all hydropower stations were assigned a uniform initial weight of 1, which treated them as homogeneous point‐source pressure factors in the river connectivity assessment. By applying the algorithm to all grid cells within the study area and spatially overlaying the overlap densities, a continuous spatial gradient surface of cumulative hydropower development pressure was ultimately generated. Assuming *x*_1_, *x*_2_, …, *x*_n_ are independent identical distribution samples extracted from the total with a distribution density function *f*, the density function $f\left(x\right)$ at a point x is estimated as $f\left(x\right)$ (Zheng et al. 2024) as follows:

$$f\left(x\right)=\frac{1}{\mathrm{nk}}\sum_{i=1}^{n}k\left(\frac{x-x_{i}}{h}\right)$$

where *f*(x) is the estimated probability density at point *x*; *k* is the weight kernel function; *h* is the threshold; *n* is the number of sample points.

Suitable habitat for *Schizothorax* spp. was reclassified using the Maximum Training Sensitivity Plus Specificity Logistic Threshold (MTSPS) to distinguish appropriate from unsuitable areas for the fish (Aidoo et al. 2022). Based on the continuous probability of occurrence P output by the model, habitats were divided into two basic categories: Suitable (P > MTSPS) and Unsuitable (P < MTSPS). Ecologically suitable areas were basins where *Schizothorax* spp. are suitable and are minimally impacted by hydroelectric power plants; non‐ecologically suitable areas were basins where there are unsuitable conditions or where these species are significantly impacted by hydroelectric power plant development (Zheng et al. 2024).

## 3 Results

### 3.1 Model Accuracy and Primary Environmental Drivers of Potential Distribution of *Schizothorax* spp.

The MaxEnt model exhibited strong predictive ability for identifying potential habitat suitability of the three *Schizothorax* spp., with AUC/TSS values of 0.955 (±0.029)/0.916 (±0.007) for *O. stewartii* , 0.936 (±0.039)/0.874 (±0.014) for *S. macropogon* , and 0.959 (±0.023) / 0.899 (±0.012) for *S. waltoni* (Table 2).

**TABLE 2:** AUC/TSS values and influential environmental factors for *Schizothorax* spp.

Species	AUC values	TSS values	Variable	Percent contribution (%)	Permutation importance (%)
*O. stewartii*	0.955 (±0.029)	0.916 (±0.007)	FA	74.6	59.2
			Bio11	7.8	24.6
			Altitude	6.7	5.2
			Bio5	3.4	1.7
			Bio14	3.2	5.6
			Slope	1.9	0.4
			Bio4	1.5	0.2
			NDVI	0.8	3.2
*S. macropogon*	0.936 (±0.039)	0.874 (±0.014)	FA	71.1	46.4
			Bio11	19.5	47.7
			Bio14	8.5	4.5
			Slope	0.6	0.8
			NDVI	0.3	0.2
			Altitude	0	0.1
			Bio4	0	0.2
*S. waltoni*	0.959 (±0.023)	0.899 (±0.012)	FA	68.1	41.2
			Bio11	18.8	49.5
			Slope	3.7	2.3
			Bio14	3.4	2.6
			Altitude	3.1	0.1
			NDVI	2.8	4.3
			Bio5	0.1	0

**FIGURE 2:**
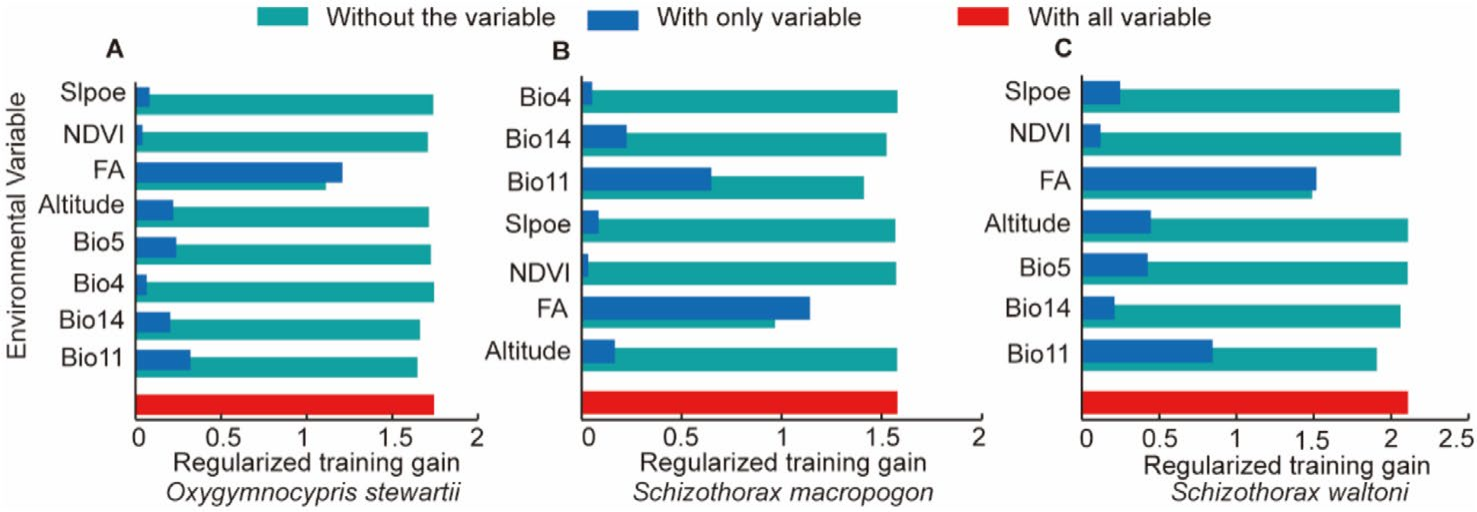
Significance of environmental factors in training data using the Jackknife test for *Schizothorax* spp.

In the MaxEnt model, environmental factors were assessed based on their percent contribution and permutation importance to identify those with the most significant influence on species habitats. The results indicate that the top two environmental factors were FA and Bio11 (Table 2). Furthermore, in combination with the Jackknife test, FA and Bio11 were identified as the key environmental elements driving geographic ranges in the MUR of *O. stewartii* (Figure 2A), for *S. macropogon* (Figure 2B), and *S. waltoni* (Figure 2C).

**FIGURE 3:**
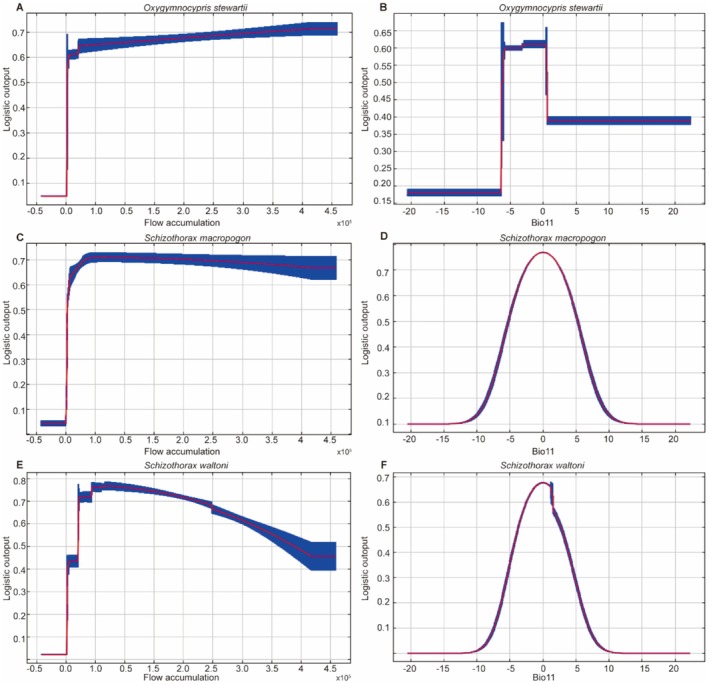
Response curve of environmental variables for *Schizothorax* spp.

Generally, environmental factors associated with a probability of occurrence greater than 0.5 are considered more conducive to species survival and reproduction. As shown in Figure 3, the optimal habitat conditions for *O. stewartii* (Figure 3A) involve an increase in flow accumulation, leading to a gradual expansion of its distribution in the MUR, ultimately reaching equilibrium. The suitable temperature range for the average temperature of the coldest quarter is between −6°C and 0.5°C (Figure 3B). For *S. macropogon* , the distribution in the MUR similarly increases with flow accumulation, stabilizing over time (Figure 3C). The optimal range for the coldest quarter's average temperature is between −5.5°C and 5.5°C (Figure 3D). In the case of *S. waltoni* , with increasing flow accumulation, its distribution gradually extends and stabilizes in MUR (Figure 3E). The most suitable range for the coldest quarter's average temperature is from −3.5°C to 3°C (Figure 3F).

### 3.2 Mapping and Planning of Suitable Habitats for *Schizothorax* spp.

**FIGURE 4:**
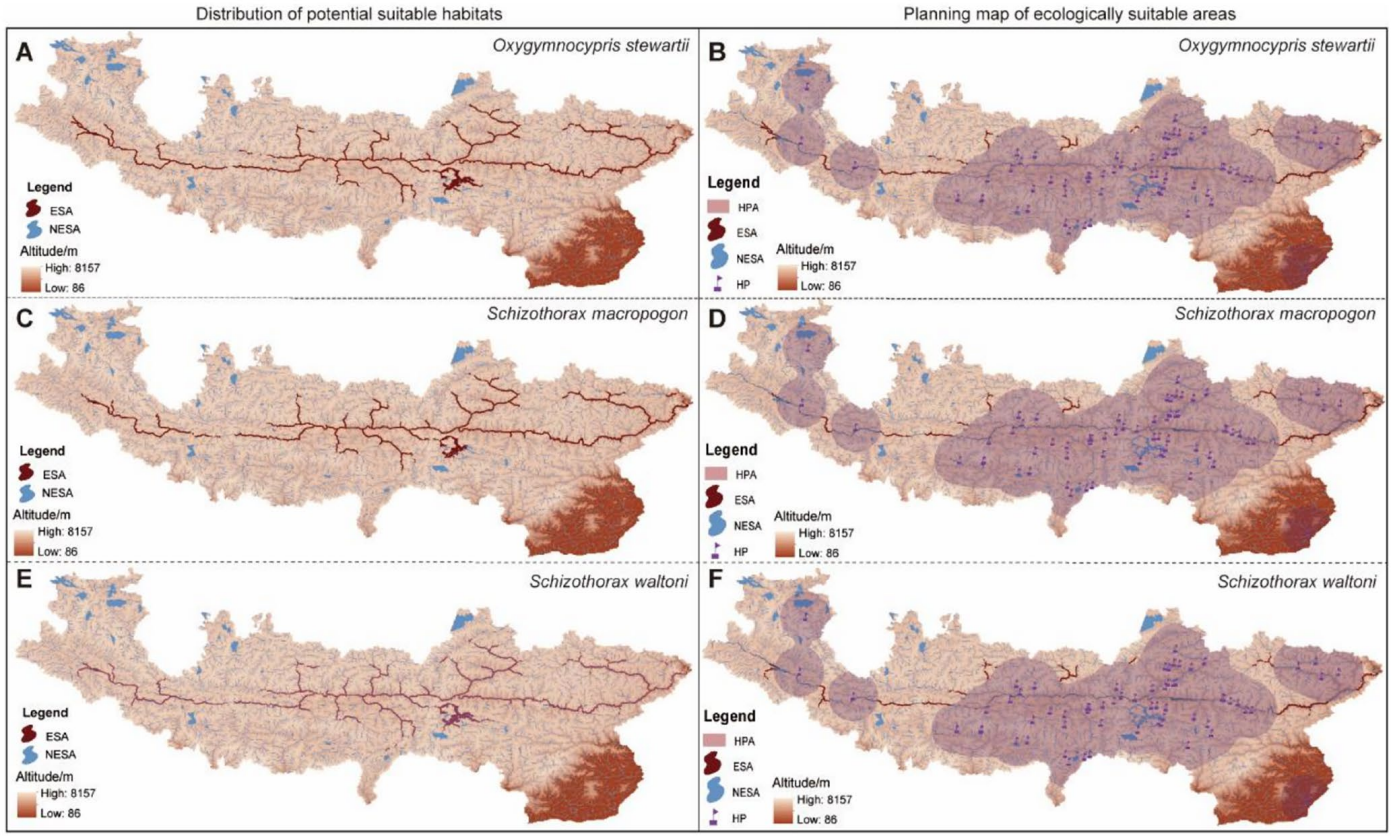
Ecologically suitable areas (left panel) and planning ecologically suitable areas (right panel) for *Schizothorax* spp. Ecologically suitable areas indicate predicted potential suitable habitats; Planning ecologically suitable areas presents predicted potential suitable habitats after accounting for hydropower‐related disturbances; ESA, ecologically suitable areas; HP, hydropower plants; HPA, influence areas by hydropower plants; NEAS, non‐ecologically suitable areas.

Ecologically suitable areas for *O. stewartii* , *S. macropogon* , and *S. waltoni* were determined to be 3147.89 km^2^ (Figure 4A), 3277.67 km^2^ (Figure 4C), and 2988.12 km^2^ (Figure 4E), respectively. The potentially ecologically suitable areas for *Schizothorax* spp. are mainly located in the MUR, including the Linzhi basin, Renbu basin, Shigatse basin, Shannan basin, Lhasa basin, Saga basin, and Miling basin of the MUR.

The KDE analysis of the hydropower station indicates that regions with more station aggregation suffer more significant impacts. Hydropower stations are primarily concentrated at the intersection of the Shigatse basin, Shannan basin, Lhasa basin, and a small portion of Miling basin, with their influence gradually decreasing outward. Beyond these core basins, the impact is relatively minimal. Under the influence of the hydropower station, *Schizothorax* spp. were primarily distributed along the river sections of Saga basin, Angren basin, and Miling basin, covering areas of 609.87 km^2^ (Figure 4B), 621.89 km^2^ (Figure 4D), and 436.46 km^2^ (Figure 4F), respectively.

### 3.3 Changes in Ecologically Suitable Areas for *Schizothorax* spp. in Future Climate Scenarios

**FIGURE 5:**
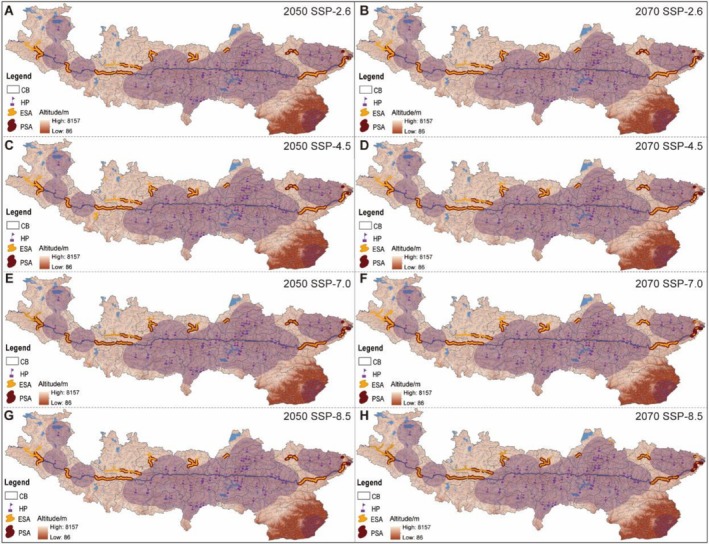
Ecologically suitable areas of *O. stewartii* under future climate scenarios. CB, county boundary; ESA, ecologically suitable areas; PAS, ecologically suitable areas under the influence of hydropower plants.

**FIGURE 6:**
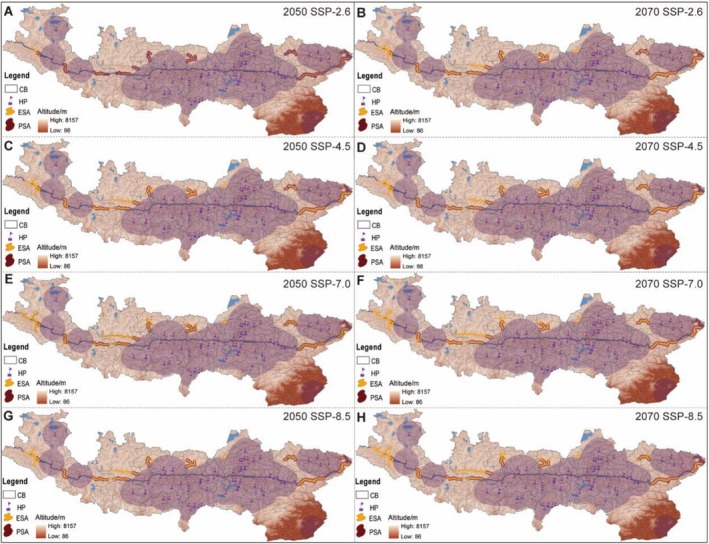
Ecologically suitable areas of *S. macropogon* under future climate scenarios. CB, county boundary; ESA, ecologically suitable areas; PAS, ecologically suitable areas under the influence of hydropower plants.

**FIGURE 7:**
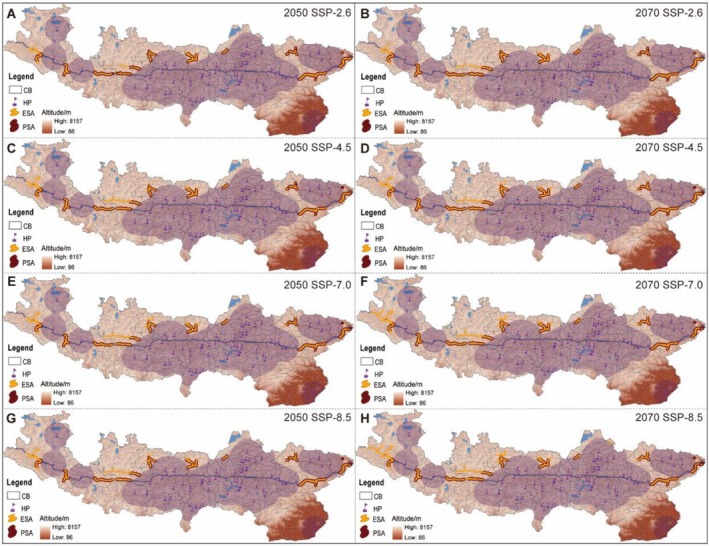
Ecologically suitable areas of *S. waltoni* under future climate scenarios. CB, county boundary; ESA, ecologically suitable areas; PAS, Ecologically suitable areas under the influence of hydropower plants.

Under projected future climate scenarios, the planning of ecologically suitable habitats for *S. macropogon* (Figure 6A–H) and *S. waltoni* (Figure 7A–H), excluding *O. stewartii* (Figure 5A–H) in the MUR are expected to increase, with expansion primarily concentrated in the upper tributaries. Among them, *Schizothorax* spp. exhibited the most significant changes in ecologically suitable areas under the SSP8.5 climate scenario for the 2070s, showing increases of approximately 12.76% (105.22 km^2^) (Figure 5H), 18.61% (154.73 km^2^) (Figure 6H), and 17.58% (135.93 km^2^) (Figure 7H), respectively. Meanwhile, under future climate scenarios, some mainstem and tributary areas downstream of the middle river reach in Linzhi basin will no longer be suitable for *Schizothorax* spp. (Figures 5, 6, 7), with *O. stewartii* (Figure 5) and *S. macropogon* (Figure 6) losing significant habitat in this basin.

### 3.4 Shifting Centers of Mass and Altitude in Potential Ecologically Habitat Areas of *Schizothorax* spp.

**FIGURE 8:**
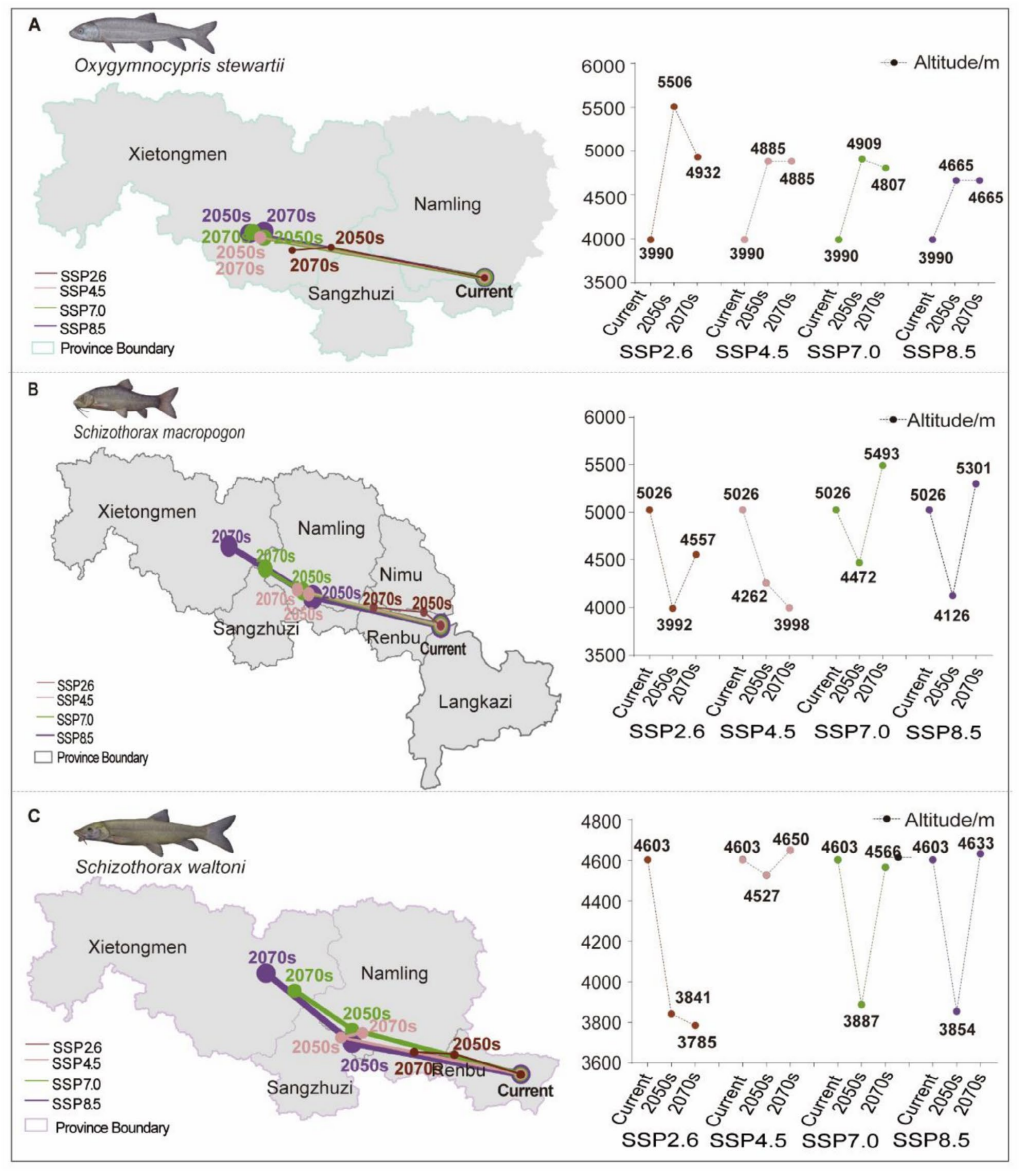
Center of mass shifts and elevation changes in suitable habitats of *Schizothorax* spp.

Owing to the irregular shape of the favorable habitats, the center of mass was used to define the core of the distribution basin, characterizing the migration of the suitable basin under different climate scenarios. The center of mass transfer is shown in Figure 8. For *O. stewartii* (Figure 8A), the center of the potentially suitable habitat basins under the present climate scenario was located at 29.3871° N, 90.5008° E. For the SSP2.6, SSP4.5, SSP7.0, and SSP8.5 climate scenarios, the center gradually shifted toward the upper tributaries of the MUR, specifically from Namling basin to the Xietongmen basin of the MUR. For *S. macropogon* (Figure 8B), the center of the potential suitable habitat under the current climate scenario was located at 29.2401° N, 90.3535° E. Under future climate scenarios (SSP2.6, SSP4.5, SSP7.0, SSP8.5), the migration of the center toward the upper basin of the MUR, specifically from Langkazi basin to the Xietongmen County basin. For *S. waltoni* (Figure 8C), the center of the potential suitable habitats under the current climate scenario was located at 29.2249° N, 90.1114° E. Similar to the other species, the center gradually migrated toward the upper rivers of the MUR, specifically shifting from Renbu County to the Xietongmen County basin under all future climate scenarios (SSP2.6, SSP4.5, SSP7.0, and SSP8.5).

In the assessment of suitable habitats elevation (Figure 8), climate change has not caused all *Schizothorax* spp. to migrate uniformly to higher elevations, but has instead triggered a reorganization of species distribution patterns. The species *O. stewartii* exhibits a pronounced upward migration trend, with the center of its potential suitable range rising significantly from the current 3990 m to an average of approximately 4907 m in the future, representing a maximum increase of 38%. In contrast, *S. macropogon* exhibits a trend of habitat reduction across most future scenarios, with significant fluctuations observed in 2070 under the high‐emission scenario. *S. waltoni* , on the other hand, demonstrates a relatively consistent downward migration pattern, though the magnitude of change is relatively small.

### 3.5 The Impact of Hydropower Plants on the Ecologically Suitable Habitats of *Schizothorax* spp.

**FIGURE 9:**
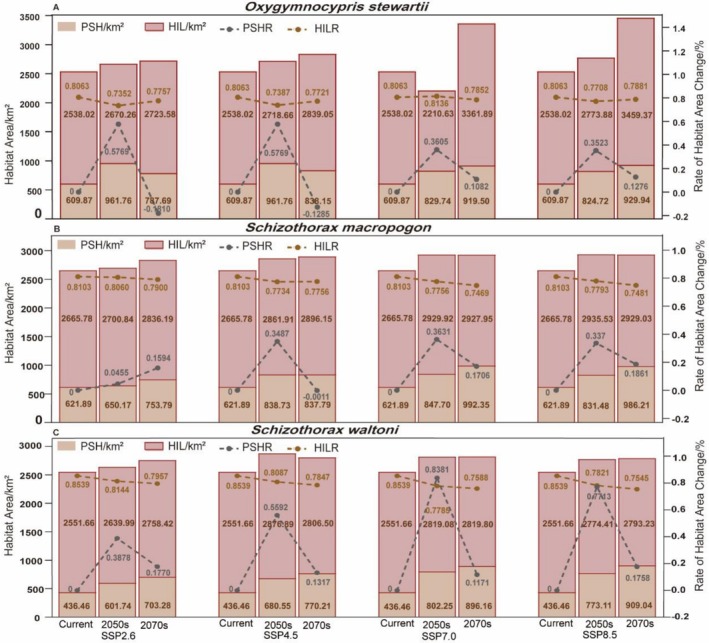
Dynamics analysis of suitable habitat area (left vertical axis, bar chart) and its change rates (right vertical axis, dashed line) for *Schizothorax* spp. under different climate scenarios. (1) HIL (Hydropower‐induced Area Loss, Dark bars): Represent the potential suitable habitat area lost due to hydropower stations pressures; (2) PSH (Projected Suitable Habitat Area, Light‐colored bars): Represent the actual suitable habitat area retained by *Schizothorax* spp. after the impact of hydropower development; (3) HILR (Hydropower‐induced Area Loss Rate, gray dashed line): Reflects the potential growth rate of suitable habitat driven by climate change; (4) PSH (Projected Suitable Habitat Area Rate, brown dashed line): Reflects the rate of habitat degradation caused by hydropower development.

This study suggests that hydropower development in the MUR has reduced the ecological suitability of habitats for *Schizothorax* spp. (Figure 9), particularly in key mainstem and tributary zones in the river's middle basins. Under different scenarios, the habitat suitability of *Schizothorax* spp. caused by hydropower plants will persist. For *O. stewartii* (Figure 9A), as the only fish losing 18.10% (174.07 km^2^) and 12.85% (123.61 km^2^) of its area in the future (under scenarios of 2070s‐SSP2.6 and 2070s‐SSP8.5), the suitable habitat faced the most severe threats. *S. waltoni* populations (Figure 9C) presented the highest potential suitability for the future, with about 83.81% (365.49 km^2^) (2050s‐SSP7.0), increasing the area of habitat suitability. However, in the medium‐to‐high‐emission or high‐emission scenario, strong adaptability will decline, as reflected in the rate of increase in suitable habitat, which will range from to 11.71% (93.91 km^2^) to 17.58% (135.93 km^2^). *S. macropogon* (Figure 9B) will also have an increase in suitable habitats in the future, reaching peak levels by the 2050s‐SSP7.0 (the growth is 36.31% (225.81 km^2^)).

## 4 Discussion

### 4.1 Key Factors Influencing the Distribution and Habitat Suitability of *Schizothorax* spp.

Habitat suitability of a species refers to its ability to survive and reproduce in an environment that fulfills its ecological requirements. It reflects the relationship between various environmental factors and species' survival strategies. For *Schizothorax* spp., FA and Bio11 were the primary environmental factors influencing distribution patterns, consistent with previous studies on fish (Mu et al. 2022; Zheng et al. 2024). This reflects the survival strategy developed by *Schizothorax* spp. in response to their long‐term adaptation to the extremely highland environment.

FA was confirmed as the most influential factor, with a significantly high contribution and replacement importance in the model (Figure 2 and Table 2). The contribution of FA reflects the dependence of *Schizothorax* spp. on hydrodynamic features and habitat connectivity. These fish exhibited different FA requirements to cope with seasonal variations in hydrology. In winter, fish move to deeper water (4–5 m) as water temperatures drop and ice forms, while they prefer to stay in shallow, slow‐moving waters for feeding and breeding in summer (Huo et al. 2013; Liu, Li, et al. 2018). In addition, FA not only determines the scale of physical space but also regulates fish spatial migration by providing specific hydrodynamic induction signals (Luo 2019). Changes in FA fundamentally reshape the continuity of river landscapes (Fausch et al. 2002). Studies indicate that flow‐velocity gradients are key signals that induce upstream migration and help *Schizothorax* spp. locate suitable habitats (Rao et al. 2025). Species such as *Schizothorax oconnori* have higher flow‐velocity thresholds and exhibit strong pro‐current swimming, whereas *O. stewartii* demonstrates superior burst‐swimming velocity (Jun et al. 2026). If the sensory signal is absent or the flow velocity exceeds their physiological power‐consumption limit (Chen et al. 2025), the fish will experience a direct result, resulting in the species losing the ability to reach spawning grounds. This sensitivity to hydrological signals represents an ecological adaptation strategy that *Schizothorax* spp. have evolved over time in high‐altitude, rapid‐flow habitats.

In this study, Bio11, which represents temperature fluctuations, significantly influences the distribution of *Schizothorax* spp., and the mean temperature of its suitable habitat in the coldest season ranged from −6°C to 5.5°C. Temperature is considered an important factor driving alpine fish distribution. This is based on the evolutionary foundation of plateau fish that have long adapted to extreme environments. Schizothoracinae fish harbor specific mutations and positive selection on hypoxia‐inducible factor (HIF‐α), conferring resistance to hypoxia and low temperature (Guan et al. 2014). Mechanistically, low temperature affects energy distribution and winter survival by inhibiting the hypothalamus‐pituitary‐thyroid (HPT) axis, reducing metabolic rate and enzyme activity (Tao et al. 2018), while climate warming induces immune stress and weakens immunity under multiple stresses (Franke et al. 2024). Freshwater fish can respond to temperature fluctuations of 0.03°C (Bull 1952), thereby interfering with growth, reproduction, and energy balance (Campana and Thorrold 2001). The study on the embryonic development of plateau endemic fish ( *O. stewartii* , *S. macropogon* ) showed that the hatching period was long (336–460 h at 10°C) and the accumulated temperature demand was high (about 2356.4 h·°C), showing a significant accumulated temperature effect (Liu, Liu, et al. 2018). Therefore, extremely low temperatures in winter or early spring not only directly kill embryos but also delay hatching, causing larvae to miss the optimal feeding time. This mechanism directly makes Bio11 a core variable. Although *S. waltoni* is highly cold‐tolerant, its reproduction is strictly regulated by water temperature (Zeng et al. 2023); the heat tolerance of its juveniles is related to the acclimation temperature, and growth rates decline significantly once the threshold is exceeded (Zhu et al. 2019). During the breeding season at water temperatures of 5°C–8°C, its respiratory rate adjusts to temperature fluctuations, demonstrating metabolic plasticity (Zeng et al. 2023); however, warming beyond the thermal equilibrium threshold induces stress and alters energy allocation. Furthermore, gonadal maturation follows a strict seasonal cycle and is finely regulated by water temperature and photoperiod (Purohit et al. 2022). Similarly, research by Ke et al. (2025) on *Gymnocypris przewalskii* in Qinghai Lake further confirms that environmental factors such as salinity and temperature significantly alter the fish's induced flow velocity and critical swimming speed, thereby limiting their distribution under specific hydrodynamic conditions. Precipitation, altitude, and other features were also shown to significantly influence alpine fish distribution patterns (Comte et al. 2013; Markovic et al. 2014; Mu et al. 2022; Zheng et al. 2024).

### 4.2 Current Distribution of *Schizothorax* spp. and Delineation of Ecological Suitability Basins

According to the survey of fish resources in the Yarlung Zangbo River, between 2017 and 2021, all *Schizothorax* spp. were successfully caught in both the middle and upper reaches of the Yarlung Zangbo River (Liu et al. 2025). This is consistent with the current MaxEnt prediction (Figure 4). Furthermore, none of these three species was caught in the lower reaches of the Yarlung Zangbo River, which supports the rationale for focusing our study area solely on the middle and upper reaches.

In this study, the ecologically suitable areas, such as the Lhasa, Shannan, and Shigatse basins (Figure 4), were classified as habitats unsuitable both currently and in the future due to the impacts of hydropower plants. This is caused by the significant drop in elevation in the MUR, and hydropower project density varies across river sections (Liu, Wang, et al. 2020). To mitigate the impacts of hydropower stations, measures such as fish passage construction (e.g., at Zangmu Hydropower Station) and restocking programs (e.g., at Jiacha and Zangmu Hydropower Stations) have been implemented. Results indicate that these measures have contributed to an increase in the populations of certain native fish species within the watershed (such as *S. younghusbandi* ), but findings suggest that the population of *O. stewartii* has not improved, likely due to factors such as feeding habits (Liu et al. 2025). However, threats posed by artificial barriers to the survival and reproduction of fish species, particularly indigenous ones, are often underestimated (Jan et al. 2025). Therefore, assessing the effects of hydropower projects on the habitat suitability of *Schizothorax* spp. is crucial for conserving the rare germplasm resources and biodiversity of the Qinghai‐Xizang Plateau.

Habitat protection measures should be implemented to ensure the sustainable development of ecologically suitable areas for *Schizothorax* spp. These include minimizing activities that fragment river habitats, such as constructing water conservancy facilities and ensuring the rational management of existing projects, reducing competition for ecological niches by regulating the release of exotic fish species, and continuously enhancing natural populations through stocking and restocking (Bosso et al. 2024; Li et al. 2025).

### 4.3 Future Changes in Ecologically Suitable Areas for *Schizothorax* spp. Under Various Climate Scenarios

Numerous studies have shown that environmental change can influence freshwater fish distribution patterns across multiple dimensions (Chen et al. 2022; Stewart et al. 2022). Under the pressure of a warming climate, species often adjust their latitudinal or altitudinal boundaries to seek optimal habitat zones and avoid unfavorable conditions, resulting in shifts in their distributional ranges (Comte et al. 2013; Telwala et al. 2013; Freeman et al. 2018). This research found a projected expansion of ecologically suitable regions (Figure 9) and shifted toward the upper basins at higher elevations of *Schizothorax* spp. (Figure 8) under future climate conditions (Figure 9). Mu et al. (2022) also found that *O. stewartii* were projected to experience a 60.0–238.3% habitat expansion (with an altitude increase of 84 m at 2090‐RCP8.5) in the future. Furthermore, lake *Schizothorax* fish (*Gymnocypris selincuoensis*) are expected to have a similar expansion (with 73 m altitude increase) (Mu et al. 2022). Sisoridae (e.g., *Glyptosternum maculatum*) in the same watershed show a declining trend in habitat quality and an upward trend in altitude (Mu et al. 2022; Zheng et al. 2024). *Schizothorax* fish in the Lancang River show a declining trend: for example, *Schizothorax lissolabiatus* habitat has declined by 19.2% since 2010 (Sun et al. 2023), and *Schizothorax lantsangensis* losses could reach up to 30.63% in the future (Xu et al. 2025). Notably, minimum temperatures in higher elevation zones increase at approximately twice (2.2°C–2.6°C of the MUR) the rate of maximum temperatures (Walther et al. 2005; Nan et al. 2005), which may accelerate the colonization of new suitable habitats by *Schizothorax* spp. following habitat loss in lower elevation basins. Moderate temperature increases are known to promote the growth rate of alpine fish species. This trend could particularly benefit *Schizothorax* spp. of the MUR, which are known for their higher temperature tolerance (up to 30°C) (Zeng et al. 2023, 2019). Therefore, the appropriate increase in temperature enhances the usability of previously unsuitable upstream habitats. However, the life feature of alpine fishes makes them especially sensitive to changes in surrounding environmental factors such as precipitation, temperature, and habitat fragmentation (Chen et al. 2022; Panja et al. 2021). For *Schizothorax* spp., such as late maturity and low reproductive rates, suggest that their dispersal is much slower than that driven by climate and environmental changes (Ding 2014; Liu 2017; Guo et al. 2019; Guo 2022).

### 4.4 Impact of Hydroelectric Projects on the Ecological Suitability of *Schizothorax* spp.

In this study, the influence of hydroelectric plants on *Schizothorax* spp. was predicted to reduce the ecologically suitable habitat for *Schizothorax* spp. by approximately 2210.63–3459.37 km^2^ across nine climate scenarios. In recent years, the MUR has experienced severe fluctuations in fish populations, particularly in basins with large numbers of hydroelectric power stations, such as the Lhasa, Shannan, and Linzhi municipal districts (Guo et al. 2019; Huo et al. 2015; Xiong et al. 2023). This suggests that hydropower development has altered habitat quality for these native species, consistent with the KDE analysis here. The development of hydroelectric power has significantly transformed the habitat watersheds of native fish and heightened the risk of exotic fish species (Ding et al. 2019; Liu et al. 2021; Luo et al. 2025). Water conservation projects alter the hydrological characteristics of rivers (Sui et al. 2014), resulting in shifts in fish population composition, disruption of gene flow within populations, blocking of migration routes, and even species extinction (Agostinho et al. 2008; Barbarossa et al. 2020; Couto et al. 2023; Ding 2014; Kano et al. 2016). For example, despite nearly 40 years of conservation efforts, five rare fish in the Yangtze River are facing imminent extinction due to the influence of dams constructed along the river's mainstem (Huang and Li 2024). Similarly, dramatic changes in flow processes in the upper sections of the Mekong River resulting from dam construction have severely reduced the reproductive window of *Schizothorax lissolabiatus* , ultimately leading to population decline due to failure to replenish the species (Ding et al. 2023). From a global conservation perspective, the Qinghai‐Xizang Plateau is classified as a biodiversity hotspot (Myers et al. 2000), and its endemic fish resources hold irreplaceable evolutionary significance (Hu et al. 2019; Li et al. 2019).

### 4.5 Limitations

This study has several limitations: (i) potential sampling bias in occurrence data, fish abundance, recruitment, or characteristics at different times (Franklin 2010), (ii) assumptions inherent in presence‐only MaxEnt modeling and KDE analysis (Araújo and New 2007), (iii) the use of a single climate model and static hydrological predictors, (iv) the lack of explicit representation of dispersal barriers and connectivity (Elith and Leathwick 2009; Franklin 2010; Grill et al. 2019; Barbarossa et al. 2020), and (v) other unmodelled ecological factors such as species interactions, flow regime alterations, and extreme climate events (Bai et al. 2025; Zheng et al. 2026), all of which may bias the results. In the future, these aspects should be further explored to yield more precise findings, thereby enabling better conservation recommendations.

## 5 Conclusion

In summary, we conducted MaxEnt to estimate potential habitat suitability zones and assess key habitat factors of *Schizothorax* spp. The results indicated that FA and Bio were essential factors influencing their distribution. Hydropower infrastructure in the watershed has significantly reduced suitable habitats for *Schizothorax* spp. Although the range of suitable habitats may expand and shift to higher elevations under future climate change, without connectivity restoration, high‐elevation habitats may remain inaccessible. To further mitigate the impact of hydroelectric plants on the habitat quality of indigenous fishes, the river basins in Saga, Angren, and Miling have been designated as new ecologically suitable areas for these species.

Based on the findings, the following recommendations are proposed to protect the *Schizothorax* spp. and its habitat in the MUR. (1) Establishment of *Schizothorax* conservation areas: Designating the basins in Angren, Saga, and Miling of the MUR, which are less affected by hydroelectric power stations, as *Schizothorax* conservation areas. All forms of development should be prohibited in these areas to safeguard the natural reproduction of *Schizothorax* spp. Additionally, efforts should be made to advance and refine artificial breeding technologies for *Schizothorax* spp., ensuring a strong technical foundation for future stocking and release programs. (2) Enhanced Monitoring Systems: developing and implementing a comprehensive monitoring system to track the status of *Schizothorax* spp. under changing climatic conditions. This is expected to provide valuable data for adaptive conservation strategies. (3) Policy and Regulatory Measures: formulating and enforcing environmental protection regulations to guarantee the valid realization of conservation measures for *Schizothorax* spp. These policies should address habitat preservation, mitigation of hydropower impacts, and sustainable fisheries management.

## Author Contributions

**Yan Zhou:** conceptualization (equal), data curation (equal), funding acquisition (equal), investigation (equal), methodology (equal), software (equal), visualization (equal), writing – original draft (equal), writing – review and editing (equal). **Qize Zheng:** conceptualization (equal), data curation (equal), formal analysis (equal), investigation (equal), methodology (equal), visualization (equal), writing – original draft (equal), writing – review and editing (equal). **Fei Liu:** conceptualization (equal), data curation (equal), formal analysis (equal), investigation (equal), methodology (equal), writing – original draft (equal), writing – review and editing (equal). **Zhaofang Han:** data curation (equal), funding acquisition (equal), visualization (equal), writing – review and editing (equal). **Hongxin Liu:** data curation (equal), investigation (equal). **Xue Wang:** data curation (equal), investigation (equal). **He Gao:** data curation (equal), investigation (equal). **Hongbo Pan:** data curation (equal), investigation (equal). **Yangyang Li:** data curation (equal), investigation (equal). **Jishun Ma:** data curation (equal), investigation (equal). **Chaowei Zhou:** data curation (equal), funding acquisition (equal), supervision (equal), writing – review and editing (equal). **Yao Li:** conceptualization (equal), methodology (equal), supervision (equal), visualization (equal), writing – review and editing (equal). **Haiping Liu:** conceptualization (equal), funding acquisition (equal), methodology (equal), supervision (equal), writing – review and editing (equal).

## Funding

This work was supported by (No. 32072980), (No. 5330500953), (No. U23A20249), (No. SWU‐XJPY202302), (No. 2024YFD1200703), (No. SWU‐KR25021) and (2025‐CX‐B011). Author Haiping Liu has received research support from the National Natural Science Foundation of China (No. 32072980), Organization Department of the CPC Central Committee (No. 5330500953), the National Natural Science Foundation of China (NSFC) Joint Fund Priority Support Program (No. U23A20249), and the Special fund for youth team of Southwest University (No. SWU‐XJPY202302). Author Zhaofang Han and Chaowei Zhou have received research support from the National Key R&D Program of China (NO. 2024YFD1200703). Author Zhaofang Han has received research support from the Double First‐Class Initiative Talent Recruitment of Southwest University (No. SWU‐KR25021). Author Yan Zhou has received research support from the Innovation and Entrepreneurship Training Program of the Xizang University (No. 2025‐CX‐B011).

## Conflicts of Interest

The authors declare no conflicts of interest.

## Supporting information


**Data S1:** ece373786‐sup‐0001‐Supinfo1.docx.

**Table S1:** Information on the distribution of *Schizothorax* spp. species.
**Figure S1:** Climate factor correlation analysis.

## Data Availability

The data that supports the findings of this study are available in the Supporting Information of this article.
