# Supplementary material for: Climate Change and Hydropower Impacts on Habitat Suitability of Endangered Schizothoracinae Fishes in the Qinghai‐Xizang Plateau
Source: Ecol Evol. 2026 Jun 18;16(6):e73786. doi: 10.1002/ece3.73786 (PMC13278813; doi:10.1002/ece3.73786)
Supplement: Supplementary file 1 — Data S1: ece373786‐sup‐0001‐Supinfo1.docx. [file ECE3-16-e73786-s001.docx]

############################################

# Model calibration using kuenm and Maxent #

############################################

# Description

## The following script helps to perform the process of model calibration using

## Maxent through the R pacakge kuenm. Model calibration consists of a series of

## steps starting in the creation of several models and ending in the selection

## of parameter settings that produce the best models. To detect which ones are

## the models with best results a robust evaluation process must be performed. In

## the example, models are selected based on statistical significance, omission

## rates based on a predefined Error, and model complexity. The metrics used here,

## are partial ROC, omission rates, and AICc, in that order.

## The main processes are performed the package kuenm from GitHub. To install

## this package see instructions in https://github.com/marlonecobos/kuenm.

# loading needed package

# assuming that you installed kuenm, load it, if not installed see

# https://github.com/marlonecobos/kuenm for instructions

library(kuenm)

# set your working directory

setwd("C:/Users/hp/Desktop/Model")

# Candidate model creation

## preparing arguments (Change "YOUR/DIRECTORY" by your pertinent directory)

occ_joint <- "Fish_joint.csv"

occ_tra <- "Fish_train.csv"

M_var_dir <- "Environ_variables"

batch_cal <- "Candidate_models"

out_dir <- "Candidate_models"

reg_mult <- seq(0.1, 4, 0.1)

f_clas <- "all"

args <- NULL # e.g., "maximumbackground=20000" for increasing the number of pixels in the bacground or

# note that some arguments are fixed in the function and should not be changed

maxent_path <- "C:/Users/hp/Desktop/Model" # where Maxent is

wait <- FALSE

run <- TRUE

## runing candidate models

kuenm_cal(occ.joint = occ_joint, occ.tra = occ_tra, M.var.dir = M_var_dir,

batch = batch_cal, out.dir = out_dir, reg.mult = reg_mult, f.clas = f_clas,

args = args, maxent.path = maxent_path, wait = wait, run = run)

# Candidate model evaluation and selection (YOU CAN DO THIS WHILE CANDIDATE MODELS ARE BEING CREATED)

## check the functions help to understand arguments

## preparing arguments (Change "YOUR/DIRECTORY" by your pertinent directory)

occ_test <- "Fish_test.csv"

out_eval <- "Calibration_Results"

threshold <- 5

rand_percent <- 50

iterations <- 500

kept <- TRUE

selection <- "OR_AICc"

parallel_proc <- 10

## runing candidate models

cal_eval <- kuenm_ceval(path = out_dir, occ.joint = occ_joint, occ.tra = occ_tra,

occ.test = occ_test, batch = batch_cal, out.eval = out_eval,

threshold = threshold, rand.percent = rand_percent,

iterations = iterations, kept = kept, selection = selection, parallel.proc = parallel_proc)

# CHECK RESULTS IN WORKING DIRECTORY AND IN THE OBJECT CREATED BEFORE.
