# Supplementary material for: Climate Change and Hydropower Impacts on Habitat Suitability of Endangered Schizothoracinae Fishes in the Qinghai‐Xizang Plateau
Source: Ecol Evol. 2026 Jun 18;16(6):e73786. doi: 10.1002/ece3.73786 (PMC13278813; doi:10.1002/ece3.73786)
Supplement: Supplementary file 2 — Table S1: Information on the distribution of Schizothorax spp. species. Figure S1: Climate factor correlation analysis. [file ECE3-16-e73786-s002.docx]

Supplementary Table 1 Information on the distribution of *Schizothorax* spp species

| **Sampling points** | **Bains** | **Longitude** | **Latitude** | **Historical Site References** | | |
| --- | --- | --- | --- | --- | --- | --- |
|  |  |  |  | ***O. stewartii*** | ***S. macropogon*** | ***S. waltoni*** |
| Shigatse | Mainstream of YZR | 89.03°E | 29.351°N | 1995 (Peng et al., 1995);  2010 (Yang et al., 2010);  2019(Li et al., 2019); - | 1995(Peng et al., 1995); 1999(Wu et al., 1999); 2010(Yang et al., 2010); 2016(Liu, 2017); 2019(Li et al., 2019); - | 1995(Peng et al., 1995); 1999(Wu et al., 1999); 2010(Yang et al., 2010); 2016(Liu, 2017); 2019(Li et al., 2019); - |
| Shigatse | Mainstream of YZR | 88.86°E | 29.32°N | - | 2017(Guo, 2022); - | 2017(Guo et al, 2022); - |
| Lhaze | Mainstream of YZR | 87.5°E | 29.11°N | 2019(Li et al., 2019); - | 2010(Yang et al., 2010); 2016(Liu, 2017); 2019(Li et al., 2019); - | 2010(Yang et al., 2010); 2019(Li et al., 2019); - |
| Xietongmen | Mainstream of YZR | 88.1°E | 29.37°N | 2019(Li et al., 2019); - | 2016(Liu, 2017); 2019(Li et al., 2019); - | 2016(Liu, 2017); 2019(Li et al., 2019); - |
| Saga | Mainstream of YZR | 85.16°E | 29.32°N | 2019(Li et al., 2019); - | 2010(Yang et al., 2010); 2019(Li et al., 2019); - | 2010(Yang et al., 2010); 2019(Li et al., 2019); - |
| Zhongba | Mainstream of YZR | 83.99°E | 29.73°N | 2019(Li et al., 2019); - | 2019(Li et al., 2019); - | 2019(Li et al., 2019); - |
| Renbu | Mainstream of YZR | 89.71°E | 29.33°N | 2019(Li et al., 2019); - | 2016(Liu, 2017); 2019(Li et al., 2019); - | 2016(Liu, 2017); 2019(Li et al., 2019); - |
| Qushui | Mainstream of YZR | 90.808°E | 29.365°N | 2022(Li et al., 2022); - | 2010(Yang et al., 2010); - | - |
| Qushui | Mainstream of YZR | 90.73°E | 29.35°N | - | - | 2017(Guo et al.,, 2022); - |
| Basumtso | Lake | 93.949°E | 30.02°N | 2022(Li et al., 2022); - | - | - |
| Nang County | Mainstream of YZR | 93.083°E | 29.071°N | 2022(Li et al., 2022); - | - | - |
| Nang County | Mainstream of YZR | 93.5°E | 29.15°N | - | 2019(Li et al., 2019); 2022(Li et al., 2022); - | 2019(Li et al., 2019); 2022(Li et al., 2022); - |
| Zedang | Mainstream of YZR | 91.783478°E | 29.274245°N | 2021(Li, 2022); - | - | - |
| Jiexu | Mainstream of YZR | 92.505°E | 29.212°N | 2021(Li, 2022); - | - | - |
| Laxu | Tributaries of YZR | 91.021°E | 30.429°N | - | - | - |
| Mozhumaqu | Tributaries of YZR | 91.9155°E | 29.7676°N | - | - | - |
| Maizhokunggan | Tributaries of YZR | 91.68°E | 29.84°N | - | 2017(Guo, 2022); - | 2017(Guo et al.,, 2022); - |
| Namling | Mainstream of YZR | 89.079°E | 29.648°N | - | 2016(Liu, 2017); - | - |
| Paizhen | Tributaries of YZR | 94.68°E | 29.47°N | - | 2017(Guo, 2022); - | - |
| Mainling | Mainstream of YZR | 94°E | 29.19°N | - | 2017(Guo, 2022); - | 2017(Guo et al.,, 2022); - |
| Mainling | Mainstream of YZR | 94.18°E | 29.23°N | - | 2019(Li et al., 2019); - | - |
| Shannan | Mainstream of YZR | 91.82°E | 29.26°N | - | - | 2017(Guo et al.,, 2022); - |
| Duilongqu | Tributaries of YZR | 90.729°E | 29.856°N | - | - | - |
| Dagze | Mainstream of YZR | 91.34°E | 29.678°N | - | - | - |
| Chabalang | Wetland | 90.598°E | 29.279°N | - | - | - |
| Gongga | Mainstream of YZR | 91.04°E | 29.295°N | - | - | - |
| Sangri | Mainstream of YZR | 92.026°E | 29.243°N | - | 2010(Yang et al., 2010); 2019(Li et al., 2019); - | 2010(Yang et al., 2010); 2019(Li et al., 2019); - |
| Caina | Tributaries of YZR | 90.802739°E | 29.36391°N | - | - | - |
| Tunba | Tributaries of YZR | 90.207994°E | 29.34476°N | - | - | - |
| Nimumaqu | Tributaries of YZR | 90.18°E | 29.379°N | - | - | - |
| Xiangqu | Tributaries of YZR | 89.088°E | 29.329°N | - | - | - |
| Nianchu River | Tributaries of YZR | 88.415°E | 29.311°N | 1999(Wu et al., 1999);  - | - | - |
| Xiabuqu | Tributaries of YZR | 88.920078°E | 29.317236°N | - | - | - |
| Dogxung Zangbo | Tributaries of YZR | 88.131°E | 29.357°N | - | - | - |
| Xiabuqu | Tributaries of YZR | 88.37°E | 29.246°N | - | - | - |
| Lhaze | Mainstream of YZR | 87.418422°E | 29.108875°N | - | - | - |
| Niyang River | Tributaries of YZR | 94.135°E | 29.736°N | - | - | - |
| Niyang River | Tributaries of YZR | 94.287°E | 29.732°N | - | - | - |
| Niyang River | Tributaries of YZR | 94.354°E | 29.651°N | - | - | - |
| Ngamring | Mainstream of YZR | 86.429°E | 29.201°N | - | 1995(Peng et al., 1995); - | 1995(Peng et al., 1995); - |
| Ngamring | Mainstream of YZR | 86.896°E | 29.477°N | - | - | - |
| Ngamring | Mainstream of YZR | 86.427°E | 29.205°N | - | - | - |
| Sakya | Tributaries of YZR | 88.384°E | 28.880°N | - | - | - |
| Sakya | Tributaries of YZR | 88.384°E | 28.884°N | - | - | - |
| Sakya | Tributaries of YZR | 88.384°E | 28.876°N | - | - | - |

Note: “-” indicates that field surveys were conducted between 2019 to 2021; YZR: the Yarlung Zangbo River

Supplementary Fig. 1.
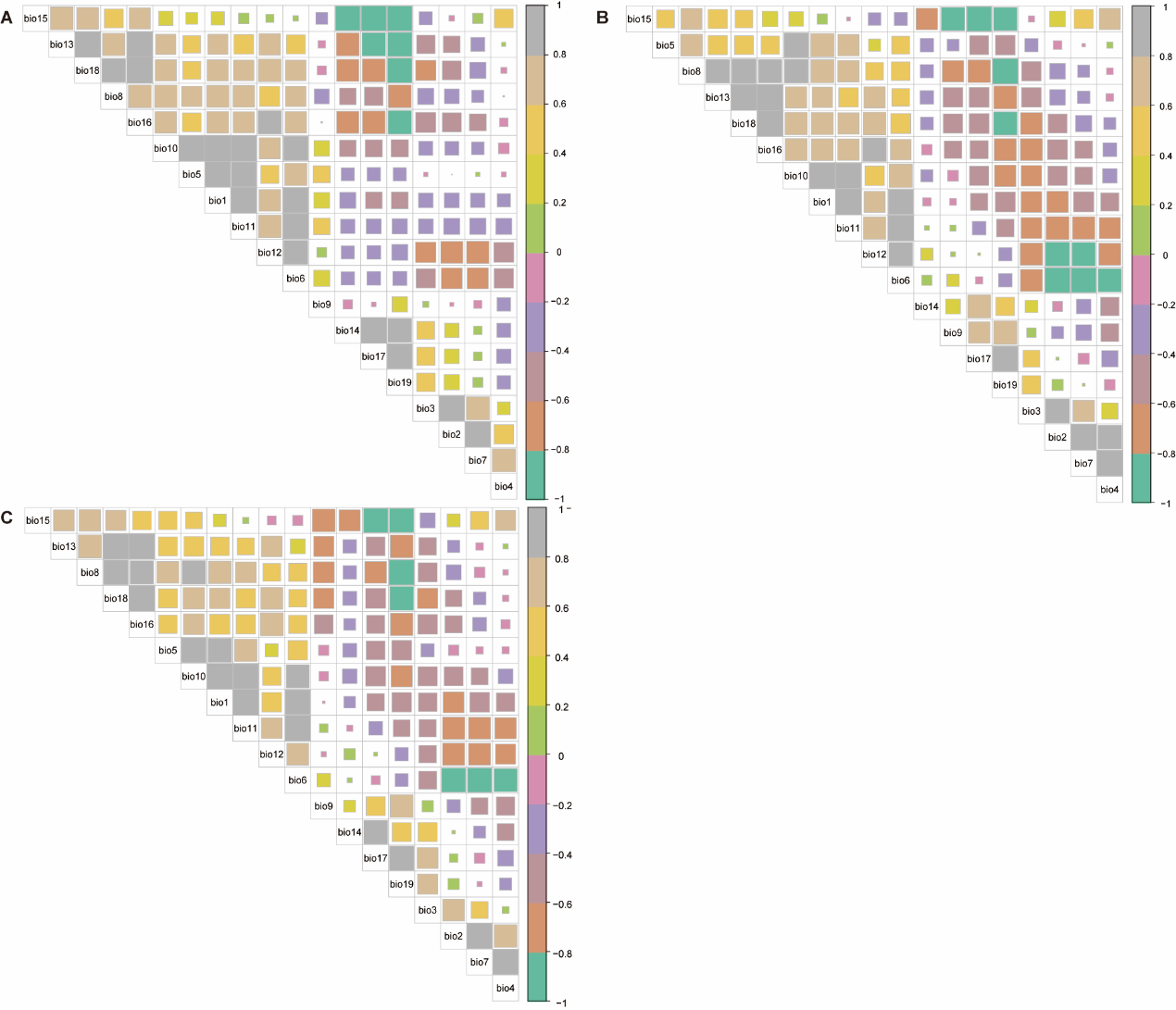


Supplementary Fig. 1. Climate factor correlation analysis

Note: A: Climate factor correlation analysis of *O. stewartii*; B: Climate factor correlation analysis of *S. macropogon*; C: Climate factor correlation analysis of *S. waltoni*
